# Restoration of Hindlimb Movements after Complete Spinal Cord Injury Using Brain-Controlled Functional Electrical Stimulation

**DOI:** 10.3389/fnins.2017.00715

**Published:** 2017-12-19

**Authors:** Eric B. Knudsen, Karen A. Moxon

**Affiliations:** ^1^School of Biomedical Engineering, Science, and Health Systems, Drexel University, Philadelphia, PA, United States; ^2^Department of Biomedical Engineering, University of California, Davis, Davis, CA, United States

**Keywords:** brain-machine interface (BMI), functional electrical stimulation (FES), paraplegia, restoration of function, encoding

## Abstract

Single neuron and local field potential signals recorded in the primary motor cortex have been repeatedly demonstrated as viable control signals for multi-degree-of-freedom actuators. Although the primary source of these signals has been fore/upper limb motor regions, recent evidence suggests that neural adaptation underlying neuroprosthetic control is generalizable across cortex, including hindlimb sensorimotor cortex. Here, adult rats underwent a longitudinal study that included a hindlimb pedal press task in response to cues for specific durations, followed by brain machine interface (BMI) tasks in healthy rats, after rats received a complete spinal transection and after the BMI signal controls epidural stimulation (BMI-FES). Over the course of the transition from learned behavior to BMI task, fewer neurons were responsive after the cue, the proportion of neurons selective for press duration increased and these neurons carried more information. After a complete, mid-thoracic spinal lesion that completely severed both ascending and descending connections to the lower limbs, there was a reduction in task-responsive neurons followed by a reacquisition of task selectivity in recorded populations. This occurred due to a change in pattern of neuronal responses not simple changes in firing rate. Finally, during BMI-FES, additional information about the intended press duration was produced. This information was not dependent on the stimulation, which was the same for short and long duration presses during the early phase of stimulation, but instead was likely due to sensory feedback to sensorimotor cortex in response to movement along the trunk during the restored pedal press. This post-cue signal could be used as an error signal in a continuous decoder providing information about the position of the limb to optimally control a neuroprosthetic device.

## Introduction

Brain-machine interfaces (BMI) have the potential to restore volitional control of paralyzed limbs following spinal cord injury (Evarts, 1968; Chapin et al., 1999; Kim et al., 2011; Hochberg et al., 2012; Jackson and Zimmermann, 2012; Collinger et al., 2013). In these approaches, neural activity during goal-directed tasks is decoded for one or more parameters of movement which can then be used as a control signal to drive an external effector (Chapin et al., 1999; Kim et al., 2011; Hochberg et al., 2012; Collinger et al., 2013) or restore movement to the affected limb directly (Ethier et al., 2012; Shenechi et al., 2014). While it is clear that BMI control of an external effector modulates neuronal responses (Ganguly et al., 2011), less is known about how these changes affect neuronal encoding of the intention to move and even less about the effect of using BMI to restore movement of the affected limb (BMI-FES). Moreover, while much of BMI work has been used to restore upper limb function in healthy animals (Ethier et al., 2012, 2015), less has been done to restore lower limb function. It is important to study restoration of lower limb function separate from that of upper limb function because the aid of visual feedback is greatly reduced when restoring lower limb function (Manohar et al., 2012).

To restore lower limb movement, investigators have mainly utilized electrical stimulation to bypass the spinal lesion. Electrical stimulation can be delivered to the musculature directly (Keith et al., 1989; Peckham et al., 2001; Daly et al., 2011; Ethier et al., 2012; Mohammed et al., 2012), inducing gross muscle contractions enabling a small yet important repertoire of functional movements such as sitting and standing. However, repetitive direct stimulation of the peripheral musculature recruits larger diameter fast-fatiguing muscle fibers before slow-fatiguing ones (Enoka, 2002; Kern et al., 2002; Russ et al., 2002; Aguilar et al., 2010), which over time may reduce the functional efficacy of peripheral stimulation. At the other end of the spectrum, intraspinal microstimulation (ISMS) targets axonal pools within the spinal cord using penetrating electrodes to elicit movements with high spatial resolution in a fatigue resistant manner (Bamford et al., 2005; Nishimura et al., 2013). While promising, electrode movement within the spinal cord could lead to damage in unconstrained subjects, precluding its clinical viability. In contrast to direct muscle stimulation and ISMS, epidural spinal cord stimulation (ES) somewhat sacrifices spatial resolution for increased stability and safety, while still benefitting from reduced fatigue by more naturally activating muscles using the intact spinal circuitry when compared with the relatively blunt approach of peripheral stimulation. Primarily, ES has been used to provide sub-threshold excitation of spinal circuitry below spinal lesions to induce weight-bearing stance and stereotypic locomotor movements (Courtine et al., 2009; Lavrov et al., 2010; Doherty et al., 2012; van den Brand et al., 2012; Gad et al., 2013). However, recent work has shown that epidural stimulation is a viable approach to improve stereotypic locomotion in spinally injured non-human primates (Capogrosso et al., 2016) and even human patients (Donati et al., 2016) in BMI tasks. However, how and whether the same approaches can be used for discrete, aperiodic movements remains an open question.

To both study the effect of FES on encoding information about the task and to evaluate the feasibility of BMI control over discrete hindlimb movements, rats were trained to press and release a pedal with their hindlimb for either a short (t<1 s) or long (1.5<t<2 s) duration in response to one of two visual cues for a reward (Knudsen et al., 2012). Recording from bilateral populations of neurons in the hindlimb sensorimotor cortex (HLSMC), the intention to press the pedal for a short or long duration was decoded while the animal still had the ability to press the pedal, when the pedal was removed, and after a complete spinal transection. Finally, the output of the decoder was used to control epidural stimulation of the spinal cord to restore a short or long duration press. Results show that BMI training produced more efficient encoding. Spinal cord injury disrupted encoding of information but HLSMC networks relearned to encode the information by changing the relative timing of neuronal responses, not their magnitude, maintaining an efficient encoding scheme after post-injury retraining. Finally, sensory information about the BMI controlled FES-induced limb movement increased the information encoded in the HLSMC. Thus, artificially generated movements of completely disconnected peripheral limbs are incorporated into the sensory experience at the level of sensorimotor cortex and are available to use in a continuous decoder for restoration of more complex movements.

## Methods

Overview: Changes in neuronal firing patterns were studied across 5 Experimental Conditions. During the Behavioral Condition animals were rewarded for pressing a pedal for the appropriate duration depending on the cue: flashing light for short (<1 s) press or solid light for long (>1, <2.5 s). During BMI-behavior (BMI-b), animals could still press the lever but were rewarded if the type of trial decoded on-line matched the cue. For BMI-only trials (BMI-o), the pedal was removed and the animal could no longer press but was rewarded if the decoded trial matched the cue. Then the animals received a complete mid-thoracic spinal cord injury, and, after recovery, were reintroduced to the task (BMI-Tx). Finally, a subset of animals was implanted with stimulating electrodes over the lumbar spinal cord and the output of the decoder was used to stimulate the spinal networks to restore specific hindlimb movements (BMI-FES). Four variables were compared across experimental condition: proportion of neurons that are responsive (change their firing rate), proportion of neurons that carry information about the task, amount of information carried by single neurons (both spike count and spike timing information) and the latency to the peak of the information.

### Animals and behavioral task

The task is similar to previously published studies (Knudsen et al., 2012, 2014; Manohar et al., 2012). However, here the animals must decode the duration of press, depending on the cue, and the output of the decoder is used to control epidural electrical stimulation in the spinal cord. Briefly, we trained adult male, Long Evans rats (*n* = 9; weight 350–400 g at time of spinal injury) to press and hold a pedal with either hindlimb for a cue-specified duration in order to earn a small drop of water for reward (0.1 mL). A flashing cue overhead indicated a short (<1 s) press, while a solid duration cue indicated a long (>1, <2.5 s) press. Animals were trained to proficiency (>80% correct cue discrimination; Knudsen et al., 2011) before surgery. All animal procedures were conducted in accordance with Drexel University Institutional Animal Care and Use Committee-approved protocols and followed established National Institutes of Health guidelines.

### Surgeries

All surgical procedures and post-operative care for rats in this study have been described in detail elsewhere (Knudsen et al., 2012; Manohar et al., 2012). In brief, all rats underwent at least 2 procedures (microelectrode implantation and complete midthoracic spinal cord injury; SCI); two animals underwent one additional EMG implantation procedure prior to SCI and 3 underwent epidural stimulator implant post-SCI. All surgical procedures were carried out under general anesthesia (2–3% isoflurane in O_2_ delivered via orotracheal intubation) and aseptic conditions.

#### Cortical and EMG implants

After training to proficiency, 4 × 4 arrays of 50 μm Teflon-insulated stainless steel microwires (MicroProbes for Life Sciences, Gaithersburg, MD) were bilaterally implanted in the infragranular layers (1.3–1.5 mm) of the rat hindlimb representation within the sensorimotor cortex (Leergaard et al., 2004). In two animals, EMG recording wires (A-M Systems, Sequim, MA, USA) were bilaterally implanted into the *vastus lateralis* and *biceps femoris* muscles of the hindlimbs and subcutaneously routed to a connector (Omnetics, Minneapolis, MN, USA) located at the animal's head just caudal to the implanted microwire array connectors.

#### Midthoracic spinal transection

After completing pre-injury recording experiments, rats received a complete T9/T10 spinal cord transection. The T7 vertebral landmark was identified after dissecting away the muscle and connective tissue surrounding the thoracic spinal column. Dorsal aspects of T9-T11 were removed and the spinal cord was transected with a scalpel blade. The surrounding spinal cord was removed to ensure no sparing of axonal tracts and sterile Gelfoam (Pfizer, New York, NY, USA) was implanted into the lesion site to discourage regeneration of neural processes.

#### Epidural electrode implantation

To perform all experiments in the current study animals would require three surgeries: (1) microwire array implants, (2) spinal transection, and (3) epidural stimulator and/or EMG implantation. It was not uncommon for animals to have bladder infections after the spinal transection and animals were treated with antibiotics. In addition, after surgery, animals might lose up to 10% of their body weight. Due to the rigors of 3 surgeries, only animals that consistently maintained a healthy weight and were free from any medical complications (mainly bladder infection) participated in the final stage of implanting the epidural stimulator and/or EMG. Prior to epidural electrode implantation, these animals were provided *ad libitum* access to water for at least 5 days before surgery. On the day of surgery, animals were anesthetized with 3% isoflurane (in O_2_) delivered through a nose cone. An incision was made along the lumbar spine and laminectomies were performed on vertebrae L3 and L5 after clearing the overlying musculature and fascia. Stimulating electrodes consisted of two wires (PFA-coated stainless-steel wire 152.4 um diameter, A-M Systems, Sequim, WA) twisted into a single stimulator, representing an anode and a cathode pair. The stimulators were inserted into the laminectomy sites (2 animals had a single stimulator, pair of wires, implanted at vertebral level L2/L3, 1 had two stimulators implanted, one at L2/L3 and one at L4/L5) and the wire was secured by suture to the musculature. Each anode/cathode pair was spaced ~2 mm apart, with the anode always placed rostral to the cathode. The wires were then routed subcutaneously to a connector at the head that was affixed to the headcap with dental acrylic. Once implant position was verified by applying trains of stimuli to elicit the proper unilateral hindlimb movements, the spinal musculature and skin were sutured closed. Animals received a 5 day course of antibiotics (enrofloxicin, 5 mg/kg) and post-operative analgesia (buprenorphine, 0.05 mg/kg).

### Single neuron discrimination

Before each recording session, single neurons were discriminated from the analog signal recorded from each microwire immediately before physiological assessment of the cells using our standard methods (Tutunculer et al., 2006; Moxon et al., 2008). Real-time spike sorting software (SortClient, Plexon Inc., Dallas, TX) captured action-potential waveform segments around a voltage threshold crossing, and sorted these in real time according to their shape. Neural signals were monitored via a computer screen using the SortClient software, an oscilloscope, and audio speakers to identify channels with candidate single units. If waveforms were greater than 3 times the root-mean square of the activity, single-neuron selection was done with a template-matching procedure based on the first two principal components of the waveforms. Most electrodes allowed us to discriminate one or two neurons (Nicolelis et al., 1997; Moxon et al., 2008). In these chronic experimental conditions, neuron waveforms are stable for hours (Nicolelis et al., 1997). For each session, neurons sorted online were re-sorted using Offline Sorter (Plexon Inc., Dallas, TX) to better isolate single neurons using the full data set. Even though we performed the spike sorting with maximum care, we conservatively acknowledge the possibility that a fraction of our neurons might represent multi-unit activity. However, the multi-unit acitivty carries information about the task and can be used in BMI task. All offline analyses (and reported cell yields) were performed using the offline-sorted datasets.

### Decoding press duration online for BMI control

To discriminate press duration as a control signal for reward, we used a simple template-matching procedure based on the real-time firing rates of all neurons recorded during the task. After each recording session, neural data were analyzed offline using a combined principal components/independent components analysis (PCA/ICA) used previously by our lab and others (Laubach et al., 1999; Manohar et al., 2012) to identify a reduced representation of the neuronal activity that best discriminated short and long press durations. Using a bin-by-bin classification approach based on the PSTH-based classifier (Foffani and Moxon, 2004), we compared single trial reduced dimensional signals with trial-average templates similar to our previously published work (Manohar et al., 2012), and found that a single independent component (IC) was sufficient to reliably decode this signal both offline (performance in BMI-b: 0.867 ± 0.01 correct; BMI-o: 0.857 ± 0.077 correct) and online (performance in BMI-b: 0.796 ± 0.011 correct, BMI-o: 0.826 ± 0.042 correct). We selected this IC using a leave-one-out approach offline using a variation of the PSTH-based classification method in which single trial population responses were compared to a set of averaged population responses (IC-PSTHs) of each trial type (templates). The minimal error between single trial and the correct (e.g., long, short, no press) template is the decoding result for that trial; the template set with the minimum error across all ICs was selected for use online. This approach was robust to the loss of neurons from day to day. Neurons lost from 1 day to the next were not included in the calculation of the population function (e.g., weights set to zero). Neurons gained were left unsorted in the online session so as to not influence the decoder, but were used in the subsequent day's decoder.

### Epidural stimulation

The optimal stimulus parameters and stimulation locations we identified previously (Doherty et al., 2012) were applied to injured animals performing BMI control after the final BMI-Tx recording session and subsequent recovery from stimulator implantation. To perform ES in the context of BMI control, a simple lookup table approach was adopted to select the stimulation parameters (stimulation duration) for the duration decoded on a given trial. To implement, the reward signal derived from a correct trial triggered the stimulator (A-M Systems Model, 2100) that was preprogrammed with the stimulation parameters (0.2 ms biphasic anode-leading pulses at 333 Hz, amplitude ~ 1–1.2 mA) determined prior to experiment onset. For each session, the animal was placed in the recording chamber, but unlike BMI-Tx where animals were sternally recumbent, we fitted each animal with a weight-supporting harness with its hindlimb secured to the amplitude sensor pedal. For a given trial, after the 1.5 s decoding period, if the decoded output coincided with the cue given, the stimulator was immediately triggered and the animal's hindlimb generated the appropriate duration movement. Stimulations were performed only every 5–10 trials to provide ample time between stimulations to ensure the spinal system had time to recover between trials.

### Data analysis

We compared changes in four variables across the four experimental conditions to those during behavioral condition: proportion of neurons that are responsive (change their firing rate), amount of information carried by single neurons (both spike count and spike timing information), proportion of duration selective neurons (carry information about the task), and the latency to the peak of the information using the data that was collected during the on-line BMI experiments, similar to our previous work (Manohar et al., 2012).

To determine if neurons were responsive, perievent time histograms (PETH) were constructed. For each neuron, PETHs were generated using 100 ms bins in a window −1,000 to 3,500 ms centered on the time of cue onset. Responsive neurons were defined as those neurons whose firing rate after cue-onset exceeded the 95% confidence interval of the firing rate before the cue. The proportion of neurons that were responsive were compared to that during behavior control using chi-squared proportion tests for each Experimental Condition.

Single neuron information was calculated separately for each neuron using the PSTH-based method (Foffani and Moxon, 2004). For each trial, the single neuron response was compared to the PETH for the short and long duration press, using a leave-one-out approach. The PETH that was closest to the single trial, in the Euclidean sense, was the decoded duration. If the decoded duration was the same as the cue, the trial was directly classified. The confusion matrix was used to convert the proportion of correct trials to bits of mutual information. Neurons that carried information (> than bootstrapping levels of information achieved when shuffling classifier labels; Laubach et al., 1999; Manohar et al., 2012) were considered duration selective. The proportion of neurons that were duration selective were compared to that during behavioral control using chi-squared proportion tests for each Experimental Condition. Spike count information was the information generated using one post-stimulus bin. To calculate spike timing information, we performed the information analysis bin-by-bin using fixed 100 ms bins while increasing the number of bins used in each step of the calculation up to 5 adjacent bins (500 ms). There were no differences in classifier performance for bin sizes less than 100 ms and therefore, 100 ms was chosen as the bins size for all subsequent on-line testing. Duration selective neurons were defined as those neurons that carried a significant amount of information about the task. The peak latency of information (seconds) was defined as the time window in which the information about the task was maximal and was calculated by increasing the size of the post-cue window and recalculating the information for each window. Latency distributions between groups were compared using the Kruskal-Wallis test.

During FES control, to evaluate when difference in information representation occurred, the relative difference in the information during the first 500 ms of the decoding window (500 ms after stimulus cue) and the first 500 ms of the stimulation window (500 ms after stimulus onset) were compared. Note that stimulations for long and short cues are identical during the first 500 ms after stimulus onset. For each neuron, we computed the ratio of information in the two epochs as an index: (I_dec_ – I_stim_)/(I_dec_ + I_stim_). Thus, as the index approaches 1, I_dec_ is increasingly greater than I_stim_ and as the index approaches −1, I_stim_ is increasingly greater than I_dec_.

Finally, we examined the population-level dynamics during the initial 500 ms of stimulation to investigate its effects on the population. The state of the network can influence the impact of encoding incoming sensory information in the motor cortex (Kaufman et al., 2016) and recent studies (Athalye et al., 2017; Gallego et al., 2017) have demonstrated that low-dimensional, shared population dynamics are of fundamental interest to motor behavior and learning. First, we performed demixed principal components analysis (Brendel et al., 2011; Kobak et al., 2016) on all trials during the decoding epoch (0–1.5 s post-cue onset) to reduce the dimensionality of our population data into a handful of components conditioned on time and trial type (short or long). Then, using the weights obtained from this epoch, we transformed neural activity surrounding the onset of stimulation into neural trajectories and examined differences in the state space mid-way through the decoding window (within 500 ms of stim start) and the evolution of the trajectory as stimulation began.

## Results

**Figure 1:**
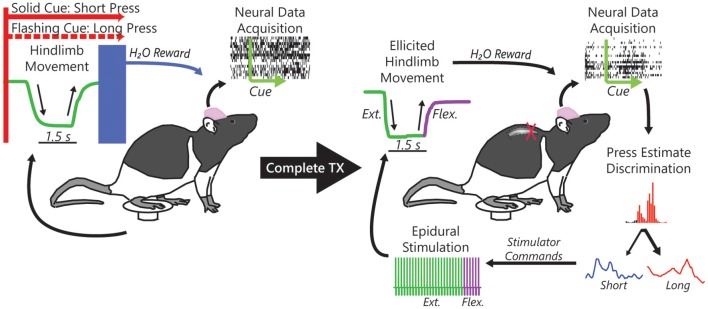
Single neuron action potentials (spikes) were recorded from populations of neurons within the rat hindlimb sensorimotor cortex while rats performed a cued hindlimb press task for a water reward. Using the acquired spikes, we formulated an online algorithm to replace the pressing behavior with neural control of the water reward. We then subjected rats to a complete midthoracic spinal cord injury (complete TX) that left the hindlimbs completely paralyzed and, after reintroduction to the task, used task-related neural activity to decode intention to press (short or long duration) in response to the cue and subsequently to control implanted epidural electrical stimulation electrodes implanted over the intact lumbar spinal cord to restore the task-dependent hindlimb movements.

Single neuron activity was recorded from 9 rats trained to produce short or long duration hindlimb presses of a pedal in response to one of two conditioned visual stimuli for reward (Figure 1). Behavioral control experiments comprised 69 recordings from the 9 rats with an average of 54 ± 18 neurons (mean ± s.d.) per session, 3,718 neurons total. In the BMI-behavior experiments we recorded 2,628 neurons over 52 sessions, with an average of 51 ± 22 neurons per session from the same 9 rats. During the BMI-only (BMI-o) experiments we recorded 2,331 neurons in 48 recording sessions (49 ± 21 neurons), again from the same 9 rats. During BC experiments, rats performed 102 ± 42 trials per session (7.7 ± 1.2 sessions per rat), 96 ± 26 during BMI-b experiments (5.8 ± 1.2 sessions per rat), and 98 ± 31 during BMI-o experiments (5.3 ± 0.87 sessions per rat).

### Hindlimb sensorimotor responses are driven by task demands Pre-TX

**Figure 2:**
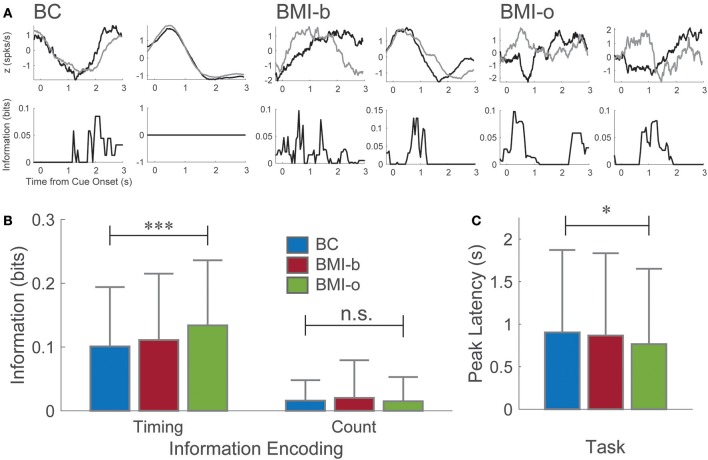
Pre-injury tasks. **(A)** Example perievent histograms from the behavioral task and the pre-injury BMI tasks. Neurons recording during the behavioral task exhibited a wide range of responses from the time of cue onset. Here, one neuron maximally encodes information about press duration at longer latency (~2 s, short press ends, long press continues) while another neuron, although clearly modulated by the movement, encodes no information about press duration. For both pre-injury BMI tasks, information is encoded maximally at shorter and shorter latencies to cue-onset. **(B)** Neurons on average encoded significantly more information about press duration (spike timing) at significantly shorter latencies **(C)** as animals moved from behavioral control to BMI-o, while there was no change in spike count information. ^*^p < 0.05; ^***^p < 0.001.

Not surprisingly, neuronal firing patterns changed as the animal learned to use the BMI control (BMI-b) and stopped pressing the pedal (BMI-o) compared to Behavioral Control (BC) as has been observed by several others (Figure 2A; Ganguly and Carmena, 2009; Ganguly et al., 2011; Long and Carmena, 2013). During BC, a majority of neurons (87.5%) were responsive post-cue onset, encoding the intention to press the pedal (Manohar et al., 2012) but the proportion of responsive neurons decreased across experimental conditions (BMI-b: 2,279 of 2,628, 86.2%; BMI-o: 1,966 of 2,331, 84.3%; chi-square proportions test: χ^2^ = 20.38, *p* < 0.001). Here, because we are comparing the responses between two different types of presses, short and long duration, we examined how the way neurons conveyed information changed. First, the proportion of neurons that carried information about press duration, duration selective neurons, increased (BC: 2,613 of 3,718 total neurons, 70.3%; BMI-b: 1,887 of 2,628, 71.8%; BMI-o: 1,720 of 2,331, 73.8%; χ^2^ = 8.715, *p* < 0.0128). Second, the amount of information carried by single neurons increased (Figure 2B; BC: 0.101 ± 0.093 bits, BMI-b: 0.111 ± 0.104 bits, BMI-o: 0.134 ± 0.102 bits; Kruskal-Wallis test, χ^2^ = 63.4, *p* < 0.0001), while the latency to the peak of information decreased across experiment conditions (Figure 2C; BC: 0.903 ± 0.97; BMI-b: 0.867 ± 0.968 s; BMI-o: 0.766 ± 0.866 s; Kruskal-Wallis test, χ^2^ = 8.24, *p* = 0.0163). Therefore, some neurons, likely those encoding other aspects of the movement, stopped becoming responsive while others increased their efficiency for encoding movement duration by responding earlier with more information.

### Complete spinal injury transiently disrupts HLSMC task encoding

**Figure 3:**
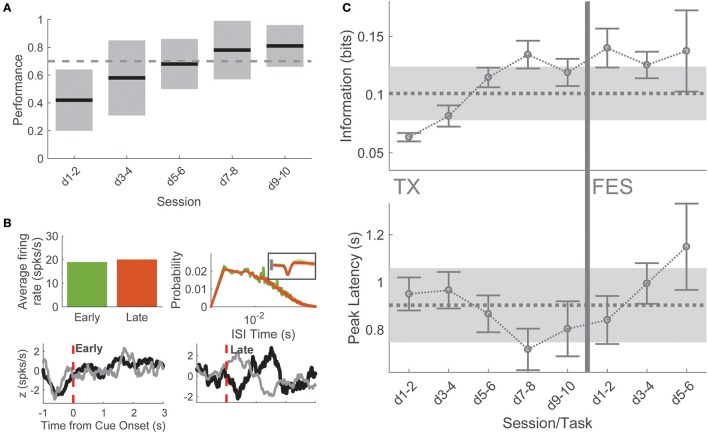
Post-TX task. **(A)** Performance in the task was initially poor for several but returned to baseline levels on days 7–8 and remained high thereafter. **(B)** Single neuron example of metrics used to compare early/late-TX information. This neuron was determined to be putatively the same sampled on days 1 and 9 of BMI-tx for one animal. There was no difference in the overall firing rate of the neuron (top left), no quantitative difference in interspike interval (top right), and no duration information encoded (bottom left). After relearning and reaching criterion performance, this neuron modified its firing such that significant information about duration was encoded at short-latency to cue onset. **(C)** Spike timing information about press duration and latency to peak information as a function of session and task. Dashed lines gray shading corresponds to behavioral control mean and standard deviation for each measure. During late BMI-tx, peak information occurred earlier than behavior on days 7–10. During BMI-FES, press duration information remained high, while latency to peak information increased severely.

Four animals were subjected to complete midthoracic spinal transection. After at least 7 days of recovery, animals were reintroduced to the BMI task and remained in the task for 7.17 ± 0.94 sessions (43 sessions total), with an average of 51 ± 12 neurons per session, similar to pre-TX recording sessions. During BMI-tx experiments, rats performed on average 74 ± 30 trials per session (10.75 ± 0.96 sessions per rat). There were no differences in the numbers of neurons recorded across each task (one-way ANOVA for number of neurons, *F* = 0.63, *p* = 0.645). On-line performance in the task initially decreased but within 6–10 days (8.2 ± 2 days after reintroduction to task) performance returned to BC levels (>70% success rate; Figure 3A).

Early sessions (“early-TX,” i.e., sessions 1–2 after reintroduction to task), when performance was poor, were marked by a large decrease in both the proportion of responsive cells (195 of 411 cells, 45.5%, χ^2^ = 47.18, *p* < 0.00001) and the proportion of duration selective neurons (153 of 411 informative, 37.2%, χ^2^ = 43.13, *p* < 0.00001) compared to behavioral control. By comparison, late sessions (“late-TX”; i.e., final two sessions before stimulation) showed a return to behavioral control levels for both proportion of responsive cells (334 of 397 cells, 84.1%, χ^2^ = 0.252, *p* = 0.616) and the proportion of duration selective neurons (292 of 397 cells, 73.6%, χ^2^ = 5.259, *p* = 0.022, Bonferroni corrected significance at *p* < 0.05/8 = 0.00625).

To better understand this recovery of on-line performance, we compared neuronal firing patterns and single neuron information on the first session after SCI and the last session before BMI-FES when performance had returned to BC levels. Neither firing rate (1-way ANOVA, firing rate × session: *F* = 1.32, *p* = 0.26) nor inter-spike interval (1-way ANOVA mean ISI × session: *F* = 0.4, *p* = 0.812) changed across TX sessions, suggesting that the change in information was not due to simple changes in firing rate (Figure 3B). This was confirmed by comparing single neuron information (*F* = 1.13, *p* = 0.3328). However, spike timing information was significantly greater during late sessions compared to early sessions (1-way ANOVA peak information; *F* = 10.74, *p* < 0.0001; *post-hoc* days 1–2 vs. days 9–10: *t* = 5.085, *p* < 0.0001). Therefore, the increase in performance during training post-TX was due to temporal changes in the neuronal responses to the cue. Interestingly, there was a decrease in latency to peak information returning to levels seen in the final BMI-o days before injury, peaking on days 7–8, which corresponds to the time point at which mean performance exceeded the 70% correct performance criterion (Figure 3C).

### Stimulation induced hindlimb movements represented in deafferented cortex

**Figure 4:**
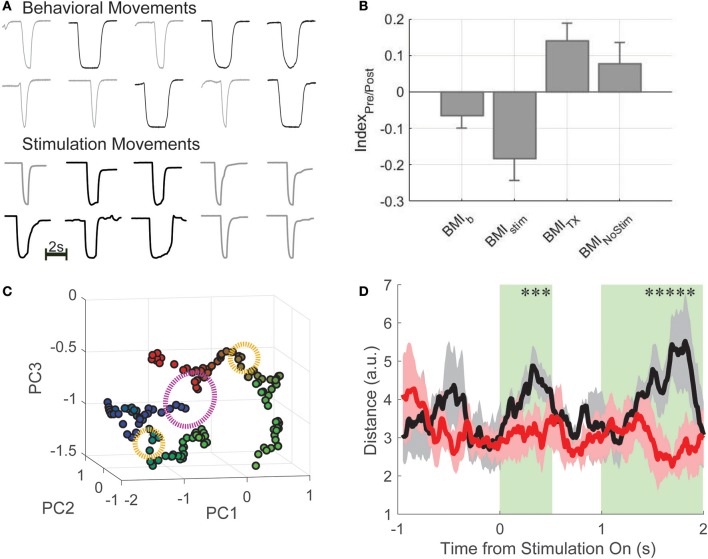
BMI-FES. **(A)** Example hindlimb amplitude traces (in volts, peak displacement, which is similar across all trials is ~2 cm; bar indicates 2 s) generated by normal behavioral movements (top) compared with artificial movements driven by the cortically-controlled epidural stimulator in the same animal after spinal injury. Qualitatively, endpoint trajectories are similar. **(B)** We computed an information index that describes the relationship between information encoded about press duration during the first 500 ms of the decoding period and the first 500 ms of the stimulation period. We found that stimulation trial indices (BMI_stim_) most closely matched those from the BMI-b condition, while post-TX trials without stimulation (BMI_NoStim_) had index values closer to BMI-tx values. **(C)** Using dimensionality reduction, we show that the state of the neural population (green clusters representing post-cue window) is different for long (blue) compared to short (red) trials, and has a marked effect on how stimulation (orange circles) affects population activity. Subsequent evolution of the neural trajectories after stimulation onset (when green turns to blue for long duration presses and green to red for short duration presses) showed that the trajectories follow different paths despite the fact the stimulation is the same (purple circle denotes 500 ms post-stimulation onset). **(D)** Euclidean distance between trajectories generated during short and long duration trials during stimulation sessions (black curve) and during non-stimulation sessions (red curve). ^*^Denotes deviation from 95% confidence interval and shaded in green.

After the final BMI-tx sessions, when performance criterion (70% true positive rate) was reached over the course of several sessions, three rats that had maintained good health and weight after injury were implanted with either a single pair of stimulating wires (*n* = 2) to elicit hindlimb extension or two pairs of wires (*n* = 1) to generate extension and flexion movements based on our previous work (Doherty et al., 2012). The output of the decoder was used to trigger delivery of appropriate current and effectively drive hindlimb movements from cortical neural activity (Figure 1). For a given trial, if the online decoder correctly classified the trial, the stimulator was triggered and the animal's hindlimb generated the appropriate movement (short or long press) and the animal was given a water reward. Stimulations (~1–1.2 mA constant current) were performed approximately every 2 min (~5–10 trials per cue type) on correctly classified trials. Paw trajectories are shown in Figure 4A for the animal with dual stimulators compared from trajectories taken from an early behavioral session from the same animal. In this session, 51 of 58 total trials were correctly decoded and stimulations were performed on 15 of these trials. For each stimulation trial, the stimulator was triggered immediately following the decoding result for the duration corresponding to the cue type delivered (e.g., short or long) and the resultant hindlimb trajectories (as quantified by a one-dimensional amplitude trace) are shown for each trial.

Having successfully generated hindlimb movements via BMI-driven epidural stimulation, the impact of the restoration of the movement on the encoding of information about the press was investigated, recording 904 neurons from 3 rats across 18 sessions (50 ± 25 per session). On average, rats performed 65 ± 12 trials per session (6 session per rat). Neither the proportion of duration selective neurons nor the single neuron information changed between late BMI-tx and BMI-FES (proportion: late-BMI-tx: 83.2%; BMI-FES: 720 of 979 neurons, 79.7%; χ^2^ = 0.299 *p* = 0.585; information: late-BMI-tx: 0.172 ± 0.108 bits, BMI-FES: 0.203 ± 0.134 bits, *t* = 1.344, *p* = 0.181). However, the latency of peak duration selectivity during BMI-FES was shifted significantly later, even later than that during BC (BC latency: 0.904 ± 0.97 s; BMI-FES: 1.116 ± 0.764 s, Wilcoxon rank-sum *Z* = 5.42, *p* < 0.0001). In fact, there were more neurons with peak information encoding during the stimulation window (>1.5 s post-cue onset) compared to the same populations of neurons during the pre-stimulation window, when the decoded information was used to determine stimulation type (stimulation: 1.138 ± 1.019 s; no stimulation: 0.881 ± 0.99 s, *Z* = 1.91, *p* = 0.056). In addition, there was on average greater single neuron information during the first 500 ms of stimulation trials compared to a comparable window during no stimulation (NO STIM) trials (stimulation trials: 0.287 ± 0.192 bits, no stimulation: 0.189 ± 0.117 bits, *Z* = 4.008, *p* < 0.0001).

To rule out that this effect was due purely to differences in applied stimulation, and to determine if these findings could be related to sensory feedback from movements above the level of the lesion in response to the restored pedal press, we computed and compared stimulation index values described above. Overall, there was a significant effect of group on index values [Figure 4B; one-way ANOVA for group; *F*_(36, 18)_ = 8.17, *p* = 2.5e-5]. BMI-b index was closest to zero suggesting the most balanced representation of information across the decoding window and movement window. Since there was no movement during BMI-tx, this task had the largest positive index values, and, not surprisingly, were significantly different from BMI-b index values (2-sample *t*-test, *t* = −3.59, *p* = 0.0004; Bonferroni corrected critical *p*-value: 0.0204). BMI-stim index values were the most negative, suggesting substantial information during the stimulation window compared to information during the decoding window. In fact, BMI-stim index values were significantly different from those of both BMI-Tx and BMI-nostim (vs. BMI-Tx: *t* = −4.28, *p* < 0.0001; vs. BMI-nostim: *t* = −3.07, *p* = 0.0025) but not different from BMI-b (*t* = −2.04, *p* = 0.0424). Results of nonparametric statistics were the same.

To understand how duration specific information was generated during the first 500 ms of stimulation when movements and stimulations for short and long duration presses were the same, we examined the state and state-space trajectories of the neuronal activity by plotting the first three Principle components as a function of time during stimulation trials (Figure 4C). Starting mid-decode (1 s after cue onset), there is a clear separation between population activity on short and long duration trials (green clusters, blue = long duration, red = short) suggesting that the intention to make a short or long press resulted in different population states. Subsequent evolution of the neural trajectories after stimulation onset (when green turns to blue for long duration presses and green to red for short duration presses) showed that the trajectories follow different paths despite the fact the stimulation is the same. To quantify this, we compared the total Euclidean distance between trajectories generated during short and long duration trials across all BMI-FES sessions during stimulation (black curve) and non-stimulation trials (red curve; Figure 4D). We found that there were significant differences between stim and no-stim distances in the initial 500 ms post-stim onset, and, predictably, when the stimulation was turned off for short duration trials yet remained on for long duration trials (t ~ 1–1.5 s). Thus, despite differences, stimulation drove the information about the task in a manner most similar to the way information was represented during BMI-b task, when animals were using BMI control but still performing the hindlimb movements.

## Discussion

Little is known about the impact of SCI and even less about the impact of BMI controlled stimulation on neural encoding. Understanding this can lead to the development of better decoders, especially adaptive decoders that compensate for these changes. Many studies have shown that learning in the BMI task change the firing rates and latencies of neural response. Here we examine how BMI learning changes the way these neurons encode information. We found that although the proportion of responding cells decreased, the proportion of neurons carrying information and the amount of information per neuron increased while the peak latency of the information decreased, demonstrating how neuronal populations become more efficient at encoding with BMI training. Therefore, the magnitude changes that we and others have observed as animals learn BMI control results in individual neurons becoming more efficient, carrying more information about the specific needs of the task, in this case, press duration.

Spinal cord injury initially decreased on-line performance but neurons reorganized over several recording sessions to restore information to levels seen during behavioral control. This was not due to an increase in firing rate nor an increase in the number of cells carrying information, which actually decreased, but rather due to an increase in spike timing information that is accompanied by a decrease in peak latency of the information during late-TX trials compared to early-TX trials. By utilizing a complete mid-thoracic spinal cord injury that completely severs the hindlimbs from supraspinal structures, we are able to study these effects without confounding information due to spared fibers and/or pathways that cross the mid-line.

Finally, functional electrical stimulation to restore hindlimb movement further changed the encoding of information. There were no changes in the proportion of duration selective neurons nor the single neuron information, however, the latency of peak duration selectivity was shifted significantly later, even later than that during behavioral control. Single neuron information was only increased during stimulation trials but not during no stimulation trials, suggested that these changes were in response to the movement. Finally, the latency to peak information during BMI tasks when the limbs did not move (BMI-o and BMI-tx) were most similar as were the latency during BMI tasks when the limbs did move (e.g., BMI-b and FES) while these pairs where highly different from each other, supporting the conclusion that this shift in latency of the peak duration of information is likely due to somatosensory information in response to the movement itself. Therefore, a decoder that adapts to sensory feedback will likely be able to better restore function than one that only considers information about the intention to move generated from cortical centers.

### The rat is a good model of BMI learning

Despite several studies devoted to developing algorithms to relate neural activity to external behaviors in order to ultimately control external effectors (Evarts, 1968; Georgopoulos et al., 1986; Chapin et al., 1999; Carmena et al., 2003; Lebedev et al., 2005; Wu et al., 2006; Velliste et al., 2008; Kim et al., 2011; Ethier et al., 2012; Gilja et al., 2012; Hochberg et al., 2012; Jackson and Zimmermann, 2012; Collinger et al., 2013; Dethier et al., 2013; Shenechi et al., 2014), to a large degree these studies have only addressed decoding forelimb behaviors to control effectors that rely on visual feedback. Yet, millions of people live with paraplegia due to spinal cord injury or other neurological injuries and have lost the ability to control their lower limbs. While much work still needs to be done to optimize lower limb prosthetics, this work represents one of the first studies of BMI after severe spinal cord injury resulting in complete paraplegia. Under these conditions, the rat model, despite being quadrupedal, makes an excellent model for understanding the impact of paraplegia on the functioning of a BMI. Additionally, few BMI studies have demonstrated the restoration of volitional control of movements by by-passing the injury and using the decoded cortical signal to restore function (Ethier et al., 2012; Nishimura et al., 2013), and fewer still have done so under pathologies that are typical in the patient population that brain-machine interfaces (BMI) seek to help (Nishimura et al., 2013; Capogrosso et al., 2016; Donati et al., 2016). Thus, in this study, we demonstrate for the first time to our knowledge a cortically-driven brain-machine interface for the restoration of skilled hindlimb function after severe spinal cord injury using epidural electrical stimulation. Further, it is one of few studies that has sought to investigate how BMI-driven artificially restored movements impact concomitant neural encoding.

### BMI training

The enhanced discrimination of the response is likely due to learning the BMI task. In a recent study of somatosensory learning, Long and Carmena observed similar increases in response magnitude and decreased baseline activity in sensory barrel field neurons that were well correlated to increasing performance in novel cross-modal sensory task (Long and Carmena, 2013). The same phenomenon was also observed during BMI learning in a motor context in which monkeys were trained under a BMI but were switched between epochs of BMI and behavioral control within single experimental sessions (Ganguly and Carmena, 2009; Ganguly et al., 2011; Knudsen et al., 2014).

While inclusion of the BMI-only task allowed our animals to learn BMI control before SCI, it is well established that human subjects can learn to modulate their neural activity after neurological injury (Kim et al., 2011; Collinger et al., 2013) or disease (Kennedy and Bakay, 1998) and it is expected that rats would be able to do this as well. Furthermore, BMI studies have demonstrated that arbitrary decoders can be learned by cortical networks (Clancy et al., 2014; Law et al., 2014). Thus, although some learning is necessary to control the stimulator took place pre-transection, much of this learning was lost post-SCI and it is likely that, had we been able to direct the animal's intentions after SCI without the intervening steps taken here, the recorded neurons would have learned to control the stimulator. Despite this “pre-training” period pre-injury, substantial additional learning was necessary for the injured rats to perform well in the task as evidenced by the fact that animals required several sessions before performance reached pre-TX levels. Moreover, this experimental design allowed comparisons of neural responses after SCI and during BMI controlled FES to those before.

### Brain-machine interface to functional electrical stimulation (BMI-FES)

Functional stimulation has been used to restore grasp in monkeys performing a reach and grasp task (Ethier et al., 2012) but that work was limited to local nerve block used to model injury and the stimulation was delivered intramuscularly. In both that work and the current study, the stimuli delivered were cortically determined and used to drive the stimulation based on *a priori* calibration of hindlimb or muscle responses determined before online experiments took place. In our study, the neural activity was decoded as either a short or long duration trial and the stimulator was triggered for the appropriate duration after the decode was complete, while Ethier and colleagues continuously modulated the output of the stimulator by linearly correlating the output of the decoder to pulse width. Investigation of a dynamic stimulation protocol is the next logical step to provide more naturalistic control of movement. For example, it will be important to address control of both dynamic postural adjustments (Bridges et al., under review) bilateral hindlimb movements (Ifft et al., 2013 for upper limbs) and sequential movements (Lu and Ashe, 2005) after injury.

The idea of a closed-loop BMI for restoration of function after neurological injury or disease is not new (Humphrey et al., 1970; Kennedy et al., 2000). For example, Jung and colleagues coupled a neuromorphic spinal stimulator that mimicked the central pattern generator to the spinal cord of a lamprey and demonstrated that bidirectional coupling produced stable and persistent oscillations (Jung et al., 2001). More recently, Nishimura and colleagues demonstrated that the local field potentials recorded from the cortex could be operantly conditioned after SCI to control a cursor on a computer screen and then used to trigger spinal stimulation to restore upper limb function (Nishimura et al., 2013). While conceptually similar to the current study, stimulation was intraspinal. The choice of intraspinal vs. epidural stimulation has not been resolved. While epidural stimulation was recently used to restore some volitional control of movement in human subjects (Angeli et al., 2014), it may be possible to use intraspinal stimulation for higher resolution control and recruitment fatigue-resistant muscle fibers (Bamford et al., 2005). Clearly more work is required to determine which approach is best and it may depend on the particular application.

### Restored movements encoded in deafferented cortex

Our results show that epidural stimulation of the spinal cord can elicit temporally-precise hindlimb movements, and that these movements are, to some degree, represented within populations of neurons in the hindlimb sensorimotor cortex. In a severe contusion model, Beaumont and colleagues demonstrated that acute FES delivered to the paralyzed hindlimbs increased afferent drive to cortex, and was coupled to increased functional outcome (Beaumont et al., 2014). Unlike that study though, it is unlikely that the phenomenon we describe here is due to trans-lesional propagation of sensory signals from the hindlimb, as histology confirmed the completeness of the spinal cord transection. Instead, we hypothesize that the biomechanical linkage between the paralyzed hindlimbs and the trunk somatosensory system above the level of the lesion is the likely source of the incoming sensory signals representing restored movements. Moreover, these signals produced organized activity that discriminated between short and long duration presses, reflecting the stimulation-driven movement sufficiently to drive the timing of the response of duration selective neurons back toward pre-injury values. We found that the BMI task drove population activity into different states throughout the decoding period. This differential state at the onset of stimulation, which placed torque on the trunk above the lesion, likely contributed to information about the movement despite the similar sensory experience during the first 500 ms when the stimulation was the same. This is similar to what we observed during behavioral task early in the movement (e.g., press lever down), yet there is substantial information encoded about press duration before any observable difference in movement kinematics (Kaufman et al., 2016). We suggest that moving forward, BMI studies attempting to restore volitional movements carefully consider the impact interventions have on cortical responses, as this integration could act as an intermediate step between no sensory feedback and full bidirectional neural interface.

## Author contributions

KM and EK conceived of the project and developed the experimental plan. EK collected the data. KM and EK analyzed the data and wrote the manuscript.

### Conflict of interest statement

The authors declare that the research was conducted in the absence of any commercial or financial relationships that could be construed as a potential conflict of interest.
